# Wound Healing Properties of Selected Plants Used in Ethnoveterinary Medicine

**DOI:** 10.3389/fphar.2017.00544

**Published:** 2017-09-06

**Authors:** Amos Marume, Gift Matope, Simbarashe Katsande, Star Khoza, Isaac Mutingwende, Takafira Mduluza, Tafadzwa Munodawafa-Taderera, Ashwell R. Ndhlala

**Affiliations:** ^1^Department of Paraclinical Veterinary Studies, Faculty of Veterinary Science, University of Zimbabwe Harare, Zimbabwe; ^2^Pharmacy Skills Training and Development Unit, EastEnd Medical Centre, Harare Institute of Public Health Harare, Zimbabwe; ^3^Department of Clinical Pharmacology, College of Health Sciences, University of Zimbabwe Harare, Zimbabwe; ^4^Pharmaceutical Chemistry Department, School of Pharmacy, College of Health Sciences, University of Zimbabwe Harare, Zimbabwe; ^5^Department of Biochemistry, Faculty of Science, University of Zimbabwe Harare, Zimbabwe; ^6^Infection Prevention and Control, School of Laboratory Medicine and Medical Sciences, College of Health Sciences, University of KwaZulu-Natal Durban, South Africa; ^7^Vegetable and Ornamental Plants, Agricultural Research Council Pretoria, South Africa

**Keywords:** ethnoveterinary, wound healing, plant extract ointments, *Erythrina abyssinica*, *Adenium multiflorum*, *Cissus quadrangularis*

## Abstract

Plants have arrays of phytoconstituents that have wide ranging biological effects like antioxidant, anti-inflammatory and antimicrobial properties key in wound management. *In vivo* wound healing properties of ointments made of crude methanolic extracts (10% extract w/w in white soft paraffin) of three plant species, *Cissus quadrangularis* L. (whole aerial plant parts), *Adenium multiflorum* Klotzsch (whole aerial plant parts) and *Erythrina abyssinica* Lam. Ex DC. (leaves and bark) used in ethnoveterinary medicine were evaluated on BALB/c female mice based on wound area changes, regular observations, healing skin's percentage crude protein content and histological examinations. White soft paraffin and 3% oxytetracycline ointment were used as negative and positive controls, respectively. Wound area changes over a 15 day period for mice treated with *C. quadrangularis* and *A. multiflorum* extract ointments were comparable to those of the positive control (oxytetracycline ointment). Wounds managed with the same extract ointments exhibited high crude protein contents, similar to what was observed on animals treated with the positive control. Histological evaluations revealed that *C. quadrangularis* had superior wound healing properties with the wound area completely returning to normal skin structure by day 15 of the experiment. *E. abyssinica* leaf and bark extract ointments exhibited lower wound healing properties though the leaf extract exhibited some modest healing properties.

## Introduction

Alternative sources of medicines like herbal medicines may soon become key components in the healthcare provision industry for both humans and animals especially in developing countries. These herbal medicines will fill a gap caused by a decrease in the number of new modern medicines being developed in the last few decades (especially in the case with anti-infectives), increasing costs, drug resistance, and side effects of modern pharmaceuticals.

Animal wounds as a result of work related, malicious, infectious or accidental damage to the skin are often associated with significant pain, malfunctioning, limited production, disability, bleeding, and death (Forrest, 1982). Wounds may be classified as open (with disruption of skin or mucous membrane continuity) or closed (no disruption of the continuity though underlying tissues will be damaged, e.g., bruises or hematomas). Most wounds, especially open wounds, are often associated with wide ranging bacterial, fungal, and viral floras. Although most of the bacterial infections can be managed using conventional antibiotics, there are several reports of multi-drug resistant bacteria (Nolff et al., 2016). Therefore, there is need for alternative therapies in wound management.

Natural products like herbal remedies have served human and animal populations in wound management for centuries due to their efficacy, accessibility, increasing scientific data on them and commercial interests (Barreto et al., 2014). Most plants or parts thereof contain phytoconstituents that have wide ranging properties like antimicrobial, anti-inflammatory, antioxidant, antipruritic, hypotensive, proliferative, hypoglycemic, and analgesic that are often key in wound management or healing (Ayyanar and Ignacimuthu, 2009; Barreto et al., 2014).

However, use of herbal medicines is increasing concerns on their safety as only about 10% are properly characterized and/or are being produced following strict quality standards (Ifeoma and Oluwakanyinsola, 2013). As plants do contain arrays of phytochemicals with significant toxic potentials to vital organs and other living tissues, evaluation of potential toxic effects of plants and their products is essential in ensuring safety (Jothy et al., 2011).

Plants such as *Cissus quadrangularis* L., *Adenium multiflorum* Klotzsch and *Erythrina abyssinica* Lam. Ex DC. have many applications in enthoveterinary medicine world over. Traditional applications of *C. quadrangularis* (Vitaceae or grape family) include managing wounds, anthrax, hematuria, elephantiasis, broken and dislocated bones, anemia, dehydration, gout, syphilis and other venereal diseases, gastrointestinal disorders, cardiovascular diseases, scurvy, ear and eye diseases and metabolic disorders (Jainu et al., 2005; Mohanambal et al., 2011; Raj and Joseph, 2011; Kalpana, 2013; Rasale, 2014; Ruskin et al., 2014; Ghouse, 2015; Gulzar et al., 2015). Apart from being useful as an arrow poison in game hunting and fishing *A. multiflorum* (Apocynaceae family) has also many ethnoveterinary medicinal uses. The uses include the management of animal wounds, boils, warts, poultry watery/bloody diarrhea, eye and ear diseases, ticks and lice and tooth decay (Neuwinger, 1996; Zorloni, 2007; Hossain et al., 2014; Dharani et al., 2015; Shukla, 2015; Marandure, 2016). *Erythrina abyssinica* (Leguminoseae family) is one of 11 species in the *Erythrina* genus that have known medicinal applications in Sub-Saharan Africa. It is used for the management of inflammation, microbial and parasitic infections, eyes infections and kidney diseases (Kone et al., 2011). Use of these plants in animal wounds have been supported by preliminary ethnobotanical surveys done in several communal areas of Zimbabwe targeting small holder farmers, as well as phytochemical screening and/or evaluations by Marume et al. (2017). Therefore, the objective of this study was to determine the *in vivo* wound healing properties of 10% w/w ointments of crude extracts from *C. quadrangularis* (whole aerial plant parts), *A. multiflorum* (whole aerial plant parts) and *E. abyssinica* (leaves and bark) using BALB/c female mice based on wound area changes, regular observations, healing skin's percentage crude protein content and histological examinations.

## Materials and methods

### Plant collection and identification

The plant materials were collected from Mberengwa, Midlands Province (*Cissus quadrangularis* L. (Vitaceae)—20°28′09.0″S 29°55′23.3″E.), Karoi, Mashonaland West Province (*Erythrina abyssinica* Lam. Ex DC. (Fabaceae)—16°49′44.1″S 29°41′19.8″E) and Buhera, Manicaland Province (*Adenium multiflorum* Klotzsch (Apocynaceae)—19°17′10.7″S 31°25′20.2″E) of Zimbabwe during the months of October–December 2016. Species identification was done by qualified botanists (Mapaura A. – Head) from the National Herbarium and Botanic Garden, Harare and University of Zimbabwe where voucher specimens were deposited. Detailed plant use in ethnoveterinary medicine are listed in Marume et al. (2017).

### Extraction and ointment preparation

Extractions were based on methods used prior and had yielded phytochemicals that are known to have wide ranging biological effects (Marume et al., 2017). Fresh whole aerial plant parts samples from *C. quadrangularis*, whole aerial plant parts of *A. multiflorum*, leaf and bark samples of *E. abyssinica* were separately oven dried at 50°C for 48 h. Dried plant materials were ground into powders and extracted (1:20 w/v) with 50% aqueous methanol in an ultrasonic bath for 1 h. The extracts were filtered under vacuum through Whatman's No. 1 filter paper. The extracts were then concentrated under pressure using a rotary evaporator at 30°C and completely dried with a freeze drier overnight. The dried crude extracts were weighed and made into ointments at 10% (w/w) in white soft paraffin.

### Animal husbandry

Thirty six albino female mice (BALB/c) aged 8–12 weeks were obtained from the Department of Livestock and Veterinary Services—Ministry of Agriculture, Mechanization and Irrigation Development, Zimbabwe for the *in vivo* wound healing. The weights of the adult female mice used ranged between 20 and 30 g with an average weight of 25.07 g. The animals were acclimatized for 5 days in the experimental laboratory with room temperature of 22 ± 5°C, relative humidity of 80 ± 10% and approximately 12 h cycles of night and day. The animals were fed on standard mouse feed with water always available through standard water bottles. Protocols and procedures were approved by the Ethics and Animal welfare sub-committee, Division of Veterinary Services, Department of Livestock and Veterinary Services—Ministry of Agriculture, Mechanization and Irrigation Development, Zimbabwe, as well as the Faculty Higher Degrees by Research (HDR) Committee, Faculty of Veterinary Science, University of Zimbabwe. The animals were handled and treated following the principles outlined in the “Guide for the Care and Use of Laboratory Animals” prepared by the National Academy of Sciences and published by the National Institutes of Health (NIH publication 86-23 Rev. 1985).

### Wound healing

#### Ointments and animals groups

Wound healing properties of the freshly made ointments of *C. quadrangularis, A. multiflorum*, and *E. abyssinica* (leaf and bark separately) crude methanolic extracts were used in the assays. White soft paraffin was used as the negative control while the positive control was 3% oxytetracycline ointment (Biotet®), a well-known effective topical antibiotic.

The animals were grouped into six groups of six animals each. The groups were as follows: Group A—*Cissus quadrangularis* stem and leaf ointment; Group B—*Erythrina abyssinica* bark ointment; Group C—*Erythrina abyssinica* leaf ointment; Group D—*Adenium multiflorum* whole plant ointment; Group E—white soft paraffin; and Group F—3% oxytetracycline ointment.

#### Wound incision and ointment applications

The dorsal skin area of the test animals was shaved prior to the incision. Wounds (10 × 6 mm average) were cut on each mouse under sterile conditions (Karodi et al., 2009; Das, 2013). Chloroform was used for anesthesia to minimize pain and stress to the animals as a result of the wounding processes. The respective ointments were applied once daily after cleaning with sterile water and gauzes till day 15 (Karodi et al., 2009; Das, 2013). Wound sizes, contractions and other changes were measured and noted every 3 days. Histological evaluations of samples collected on day 15 were blindly conducted by a veterinary pathologist (Dr. Ellen Mwandiringana—Preclinical Department, Faculty of Veterinary Sciences, University of Zimbabwe, MP167, Mt. Pleasant Harare, Zimbabwe) and a histologist (Josephine Tendayi Chidaushe—Department of Anatomy, College of Health Sciences, MP167, Mt. Pleasant, Harare, Zimbabwe). All animal tissue samples collected were preserved for future use in formalin.

#### Protein content

The crude protein content was estimated by titrimetric determination of nitrogen content following Kjeldahl digestion of the skin samples in sulfuric acid (H_2_SO_4_) using copper (Cu) catalyst (Lynch and Barbano, 1999; Magomya et al., 2014). Oven dried skin samples were cleaned using scalpel blades to remove furs and then powdered. Between 0.05 and 0.09 g of the analytical samples where digested at 300°C with 10 ml concentrated H_2_SO_4_, 2.8 g Na_2_SO_4_ and CuSO_4_ to a final blue/green color. After cooling and dilution with water, 40% w/v NaOH was used to neutralize the solution and the released NH_3_ was collected in boric acid-indicator solution which was then titrated against a standard 0.125M H_2_SO_4_. The respective titer values, sample mases and standard acid concentration were used to calculate percentage nitrogen content. The nitrogen content was then converted to percentage protein content estimates using a factor (6.25) (Lynch and Barbano, 1999; Magomya et al., 2014).

#### Chemical fingerprinting

The qualitative screening of alkaloids was done by dissolving the extracts in dilute hydrochloric acid. After filtration the filtrates we individually treated with the Wagner's reagent (iodine in potassium iodide). Brown/reddish precipitates indicated the presence of alkaloids. Saponins were detected based on the froth's test, were the extracts were shaken individually in distilled water for 15 min. Formation of 1 cm layer of foam indicated the presence of saponins. Tannins were detected based on the ferric chloride test. The extracts were treated individually with few drops of neutral ferric chloride solution and a bluish black color indicated the presence of tannins or phenolic nucleus. The lead acetate test was used to detect flavonoids were after individually treating the extracts with a few drops of lead acetate solution; a yellow precipitate indicated the presence of flavonoids. The infrared spectra of the clean crude extracts of *A. multiflorum, C. quadrangularis* and *E. abyssinica* leaves were done on compressed it tablets using PerkinElmer Spectrum Version 10.5.2.

### Statistical analysis

Data on wound contraction percentage area changes and crude protein content across the groups was analyzed using regression models, ANOVA and *post-hoc* LSD at *p* = 0.05 significance level. The Statistical Package for the Social Sciences (SPSS) Version 21 was used for all data analysis.

## Results

**Figure 1:**
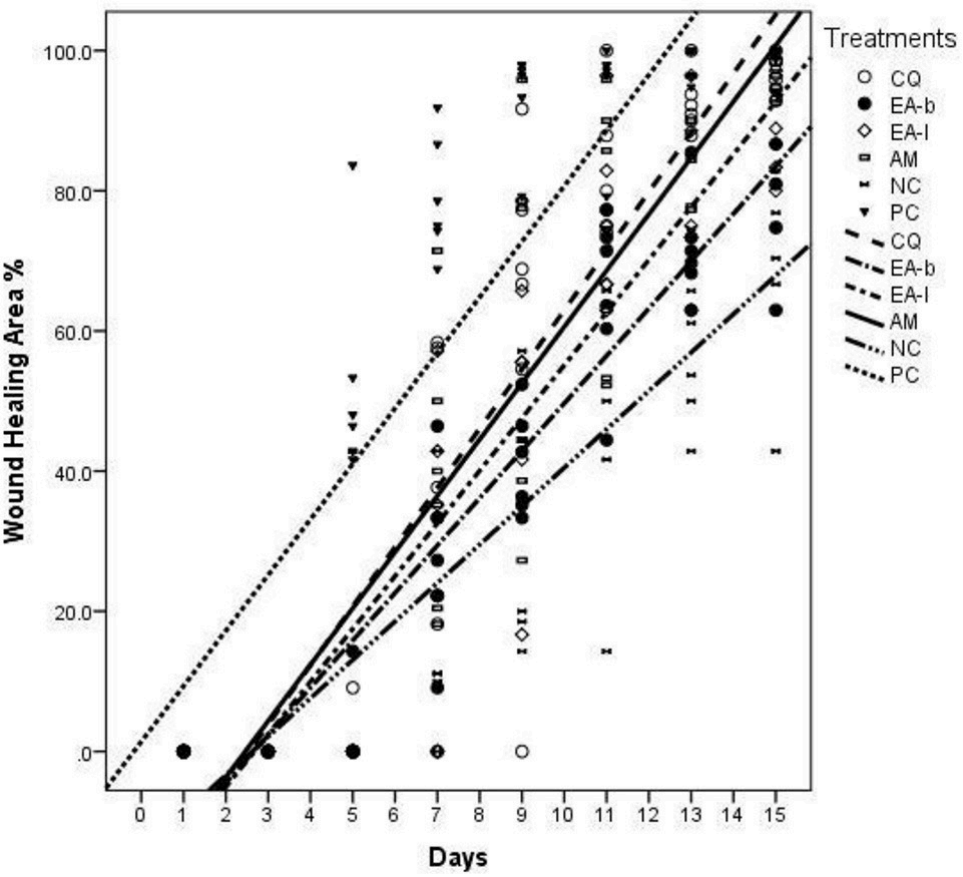
Wound healing properties as regression plots of the wound area percentage change over time as effected by ointments of three plants used in ethnoveterinary practices. CQ, *Cissus quadrangularis* whole aerial plant parts ointment; EA-b, *Erythrina abyssinica* bark ointment; EA-l, *Erythrina abyssinica* leaf ointment; AM, *Adenium multiflorum* whole aerial plant parts ointment; NC, negative control (white soft paraffin); PC, positive control (3% oxytetracycline (Biotet®) in white soft paraffin).

**Figure 2:**
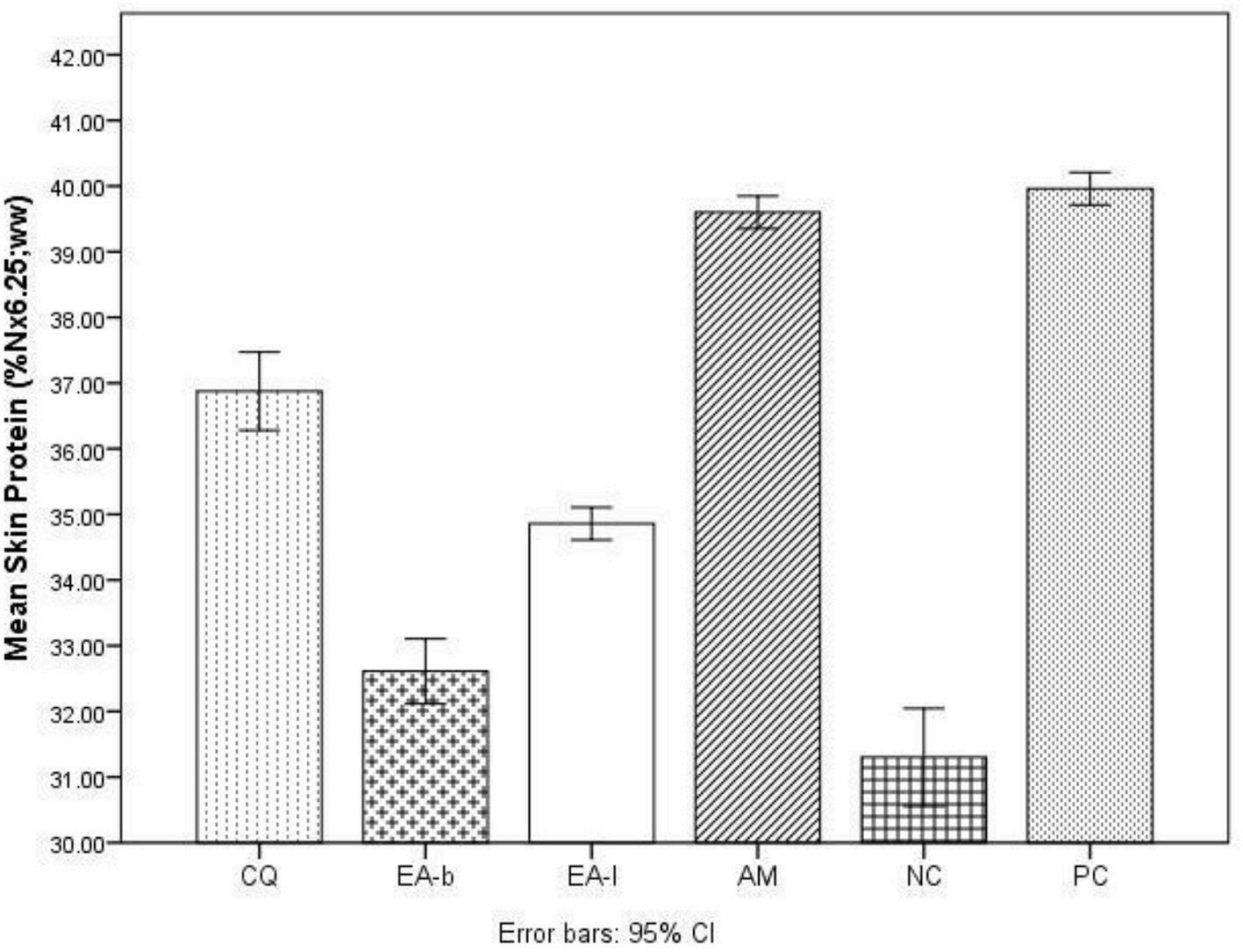
Wound healing properties as percentage crude protein content of skins taken from the healing/healed wound area as effected by ointments of three plants used in ethnoveterinary practices. CQ, *Cissus quadrangularis* whole aerial plant parts ointment; EA-b, *Erythrina abyssinica* bark ointment; EA-l, *Erythrina abyssinica* leaf ointment; AM, *Adenium multiflorum* whole aerial plant parts ointment; NC, negative control (white soft paraffin); PC, positive control (3% oxytetracycline (Biotet®) in white soft paraffin).

The results for the wound healing parameters followed in the study are presented in Tables 1–3 as well as Figures 1, 2. From Table 1 it is deduced that *C. quadrangularis* and *A. multiflorum* ointments led to wound changes and healing similar to that of the positive control over the 15 day period. On the same period *E. abyssinica* leaves and *E. abyssinica* bark ointments did not show notable differences from the negative control. In Table 2, the Positive control showed highest change in wound healing throughout the study period as compared to the rest of the treatments (*P* < 0.05). *Cissus quadrangularis* and *A. multiflorum* treatments had significantly higher wound healing effect as compared to *E. abyssinica* treatments (*P* < 0.05). However, *E. abyssinica* treatments showed significantly (*P* < 0.05) higher wound healing effects as compared to the negative control after 15 days. The effects of *C. quadrangularis* and *A. multiflorum* treatments were not significantly different after 15 days (*P* > 0.05). The negative control had the least wound healing area after 15 days as compared to the rest of the treatments (*P* < 0.05). The regression plots (Figure 1) of the wound healing area percentage change over the period of 15 days showed that all the extracts have significant percentage change of wound size as compared to the negative control. The plots also show that the positive control had the greatest rate of wound healing properties followed by *C. quadrangularis, A. multiflorum, E. abyssinica* leaves, and *E. abyssinica* barks treatments, respectively. All the regression models as shown in the plot, explained more than 70% of the variability of the wound healing area around the means. Protein content (Figure 2) in the groups treated with *C. quadrangularis* and *A. multiflorum* was found to be significantly high and comparable to that of the positive control especially the *A. multiflorum* group. *Erythrina abyssinica* leaves had considerably high protein content and *E. abyssinica* bark group had somewhat higher protein content relative to that of the negative control. As shown in Table 3, *C. quadrangularis* treated group exhibited normal and complete healing; *E. abyssinica* ointment did not show significant healing the bark ointment especially exhibited healing effects that were weaker than the negative control. *E. abyssinica* leaf ointment were somewhat better than the negative control. *A. multiflorum* ointment exhibited some healing properties better that the negative control.

**Table 1:** Wound healing properties as observations in wound changes over time as effected by ointments of three plants used in ethnoveterinary practices.

**Days**	**Treatments**					
	**PC**	**NC**	**AM**	**CQ**	**EA-l**	**EA-b**
0	Incision	Incision	Incision	Incision	Incision	Incision
2–3	Wounds secreting fluid	Wounds secreting fluid	Wounds secreting fluid	Wounds secreting fluid	Wounds secreting fluid	Wounds secreting fluid
4–6	Signs of dryness and/or contraction	No signs of dryness	Signs of dryness and/or contraction	Dryness and/or contraction	No signs of dryness	No signs of dryness
7–9	Signs of contraction	Signs of dryness	Contractions	Contractions	Signs of dryness	Dryness and/or contraction
10–12	Regeneration hair growth around lesion	Signs of contraction	Regeneration hair growth on lesion	Regeneration	Contraction	Contraction
13–15	Epithelial healing hair growth on lesion	Regeneration	Epithelial healing hair growth on lesion	Epithelial healing hair growth on lesion	Regeneration	Regeneration

*CQ, Cissus quadrangularis whole aerial plant parts ointment; EA-b, Erythrina abyssinica bark ointment; EA-l, Erythrina abyssinica leaf ointment; AM, Adenium multiflorum whole aerial plant parts ointment; NC, negative control (white soft paraffin); PC, positive control (3% oxytetracycline (Biotet®) in white soft paraffin)*.

**Table 2:** Wound healing properties as percentage wound area changes over time on treated patches as effected by ointments of three plants used in ethnoveterinary practices.

**Treatment**	**DAY 1**	**DAY 3**	**DAY 5**	**DAY 7**	**DAY 9**	**DAY 11**	**DAY 13**	**DAY 15**
CQ	0	0	1.5^b^ ± 1.51	34.2^b^ ± 9.25	59.8^b^ ± 12.97	82.4^a^ ± 4.07	92.5^ab^ ± 1.71	98.3^ab^ ± 0.55
EA-b	0	0	2.4^b^ ± 2.38	26.8^b^ ± 5.11	41.0^bc^ ± 3.03	65.1^cd^ ± 4.86	71.8^d^ ± 3.08	82.1^d^ ± 4.88
EA-l	0	0	0^b^	26.2^b^ ± 9.49	52.2^b^ ± 8.71	75.0^bc^ ± 5.18	78.3^cd^ ± 4.93	88.7^cd^ ± 2.82
AM	0	0	0^b^	39.2^b^ ± 8.09	60.4^b^ ± 11.11	73.3^c^ ± 7.94	87.6^bc^ ± 3.88	95.8^bc^ ± 1.14
NC	0	0	7.1^b^ ± 7.14	18.1^b^ ± 6.67	34.5^c^ ± 7.76	48.4^d^ ± 8.59	58.0^e^ ± 4.68	70.1^e^ ± 6.00
PC	0	0	52.7^a^ ± 6.43	79.1^a^ ± 3.49	93.7^a^ ± 2.97	94.8^a^ ± 3.15	98.5^a^ ± 0.95	99.8^a^ ± 0.21

*Superscripted letters in the same row with different superscripts are significantly different (P < 0.05)*.

*CQ, Cissus quadrangularis whole aerial plant parts ointment; EA-b, Erythrina abyssinica bark ointment; EA-l, Erythrina abyssinica leaf ointment; AM, Adenium multiflorum whole aerial plant parts ointment; NC, negative control (white soft paraffin); PC, positive control (3% oxytetracycline (Biotet®) in white soft paraffin)*.

**Table 3:** Wound healing properties as histological observations on the wound area after 15 days of treatment as effected by ointments of three plants used in ethnoveterinary practices.

***Cissus quadrangularis* whole aerial plant parts ointment (Images 28, 29, and 30)**	***Erythrina abyssinica* bark ointment (Images 31, 32, 39, and 40)**	***Erythrina abyssinica* leaf ointment (Images 33 and 34)**	***Adenium multiflorum* whole aerial plant parts ointment (Images 37 and 38)**	**3% Oxytetracycline (in white soft paraffin) ointment—Positive control (Images 35 and 36)**	**White soft paraffin—negative control (Images 41 and 42)**
Completely normal skin structure i.e., epidermis and dermis Epithelialization complete Keratinization present and abundant Numerous hair follicles and sebaceous glands present in dermis and hypodermis Collagen fibers exhibited mature arrangement	Epithelialization incomplete Absence of keratinization Evidence of inflammation in the dermis noted Absence of collagen and skin appendages noted	Epithelialization complete Keratinization present Collagen fibers present exhibiting immature arrangement Absence of skin appendages noted Granulation tissue, scab like tissue shown	Epithelialization complete Skin not yet keratinized New immature collagen fibers noted	Epithelialization complete Keratinization scanty but present Skin appendages present in the dermis and hypodermis New immature collagen fibers present	Epithelialization complete Keratinization present New immature collagen fibers present Absence of skin appendages
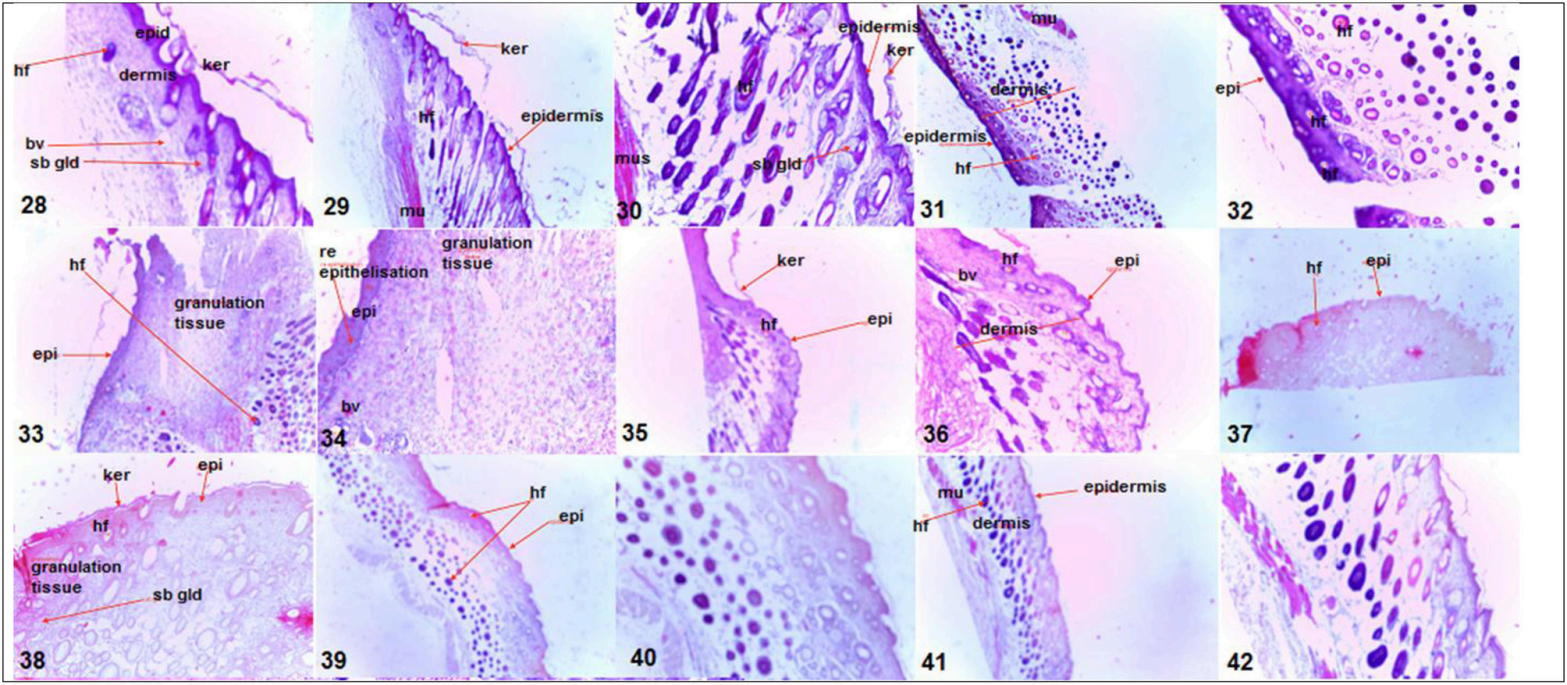					
bv, blood vessel; hf, hair follicle; epid, epidermis; ker, keratin layer; sb gld, sebacious gland; mu, smooth muscle.					

The ointments of *C. quadrangularis* and *A. multiflorum* exhibited comparable wound healing properties to the positive control (3% oxytetracycline ointment). The ointment of *C. quadrangularis* exhibited the best wound healing properties compared to the rest considering the parameters followed in this study including the positive control (Figure 1). *Erythrina abyssinica* leaf and bark ointments exhibited wound healing properties comparable to those of the negative control used with observations failing to distinguish them from the negative control (Table 1). When analysing the wound area, it was however, observed that the ointments of *E. abyssinica* leaf and bark performed better than the negative control (Table 2 and Figure 1). The histological observations (Table 3) highlighted the strong healing properties of *C. quadrangularis* ointment relative to all the other ointments. As highlighted above, *C. quadrangularis* ointment exhibited better wound healing properties than the positive control with skin and appendages returning to normal by day 15 (Table 3). *Adenium multiflorum* ointment, though exhibited good wound healing properties, it was inferior to *C. quadrangularis* ointment and the positive control used. *Erythrina abyssinica* bark ointment exhibited very poor wound healing properties as compared to the negative control. The leaf ointment of *E. abyssinica* exhibited somewhat better wound healing properties relative to the negative control.

## Discussion

Wounds are common in both humans and animals as a result of physical, chemical or thermal injuries (Barreto et al., 2014). The tissue damages if not managed often lead to chronic inflammation and secondary infections, further damaging surrounding tissues. With production animals untreated wounds may lead to significant losses due to reduced productivity as well as death.

Extracts of plants such as *C. quadrangularis, A. multiflorum*, and *E. abyssinica* have been used to manage different types of wounds. Their uses over time have thus proven efficacy and safety. In the management of wounds there are general properties which herbal remedies should possess like controlling/managing secondary infections and inflammation and promoting tissue regeneration among other healing properties. Damages associated with injury may involve the epidermis, local vasculature, dermis and possibly other underlying tissues and this usually kick starts various wound healing processes (Daunton et al., 2012). The ideal wound healing process usually follows the sequence: restoration of barrier functions of the skin e.g., with crust derived from platelets, fibrin and other blood components; handling of any invading microorganisms and other foreign debris; restoration of vasculature and tissues and remodeling of a tissue structure similar to the one damaged (Daunton et al., 2012). Often scaring occurs especially with serious and/or deep wounds where the tissue structure replacing the damaged is different in all aspects including functions. The sequences described fits in the four phases of wound healing i.e., hemostasis (immediate), inflammation (1st–4th day), proliferation or re-epithelialization/contraction (4th–21st day) and remodeling (21st day to 2 years) (Li et al., 2007; Orsted et al., 2011; Daunton et al., 2012; Soni and Singhai, 2012). Some authors however have compressed the phases of wound healing in three phases inflammatory (including clotting), proliferative and remodeling (Wild et al., 2010; Mendonca, 2012).

Herbal extracts often contain numerous molecules that are important in or as signaling molecules, lubrication, aids to proliferative process, wound contraction, cofactors, antioxidants, radical scavenging, anti-infectives, and nutrients (Raina et al., 2008). Extracts of *C. quadrangularis* were found to contain a range of molecules (Chanda et al., 2013; Marume et al., 2017) including flavonoids, tannins, glycosides, alkaloids, saponins, steroids, triterpenoids, etc. (see Table 4) known to have significant biological effects which include antioxidants, radical scavenging, antimicrobial and anti-inflammatory effects (Mohanambal et al., 2011; Kalpana, 2013; Marume et al., 2017). It also contains several elements e.g., zinc, iron, copper, manganese, etc. which are known to be essential as or in co-enzymes/factors, immunity, anti-inflammatory responses, proliferative process, etc. key aspects in wound healing (Sen and Dash, 2012; Marume et al., 2017). *Adenium multiflorum* like other members of the genus contains phytoconstituents that have toxic effects as well as key pharmacological properties in wound healing like antimicrobial, radical scavenging and anti-oxidant properties. These compounds include alkaloids, glycosides, tannins and flavonoids as well as essential trace elements (Sharma et al., 2015; Marume et al., 2017). *Erythrina abyssinica* leaf and bark methanolic extract was also shown to contain flavonoids and total phenolic compounds as well as several trace elements although in relatively lower amounts relative to *C. quadrangularis* (Marume et al., 2017). This may explain the weak wound healing effects observed and justify possible future evaluations of ointments of *E. abyssinica* extracts.

**Table 4:** Qualitative phytochemical screening of extracts of *Cissus quadrangularis* (CQ), *Erythrina abyssinica leaves* (EAl) and barks (EAb), and *Adenium multiflorum* (AM).

	**Flavonoids**	**Saponins**	**Tannins**	**Alkaloids**
CQ	+	+	+	+
EAl	+	−	−	+
EAb	+	+	+	+
AM	+	+	−	+

The presence of all phytoconstituents known to be biologically active in the extract of *C. quadrangularis* could help explain its efficacy. Marume et al. (2017) reported presence of higher levels of flavonoids and total phenolic compounds; better radical scavenging, antioxidant properties and protein precipitating properties and high levels of some trace elements known to promote wound healing in *C. quadrangularis* extracts relative to the other plants studied. In the same study *Adenium multiflorum* extract's profile followed that of *C. quadrangularis* and *E. abyssinica* exhibited the weakest which may also explain the weakest wound healing properties it exhibited in this present study.

**Figure 3:**
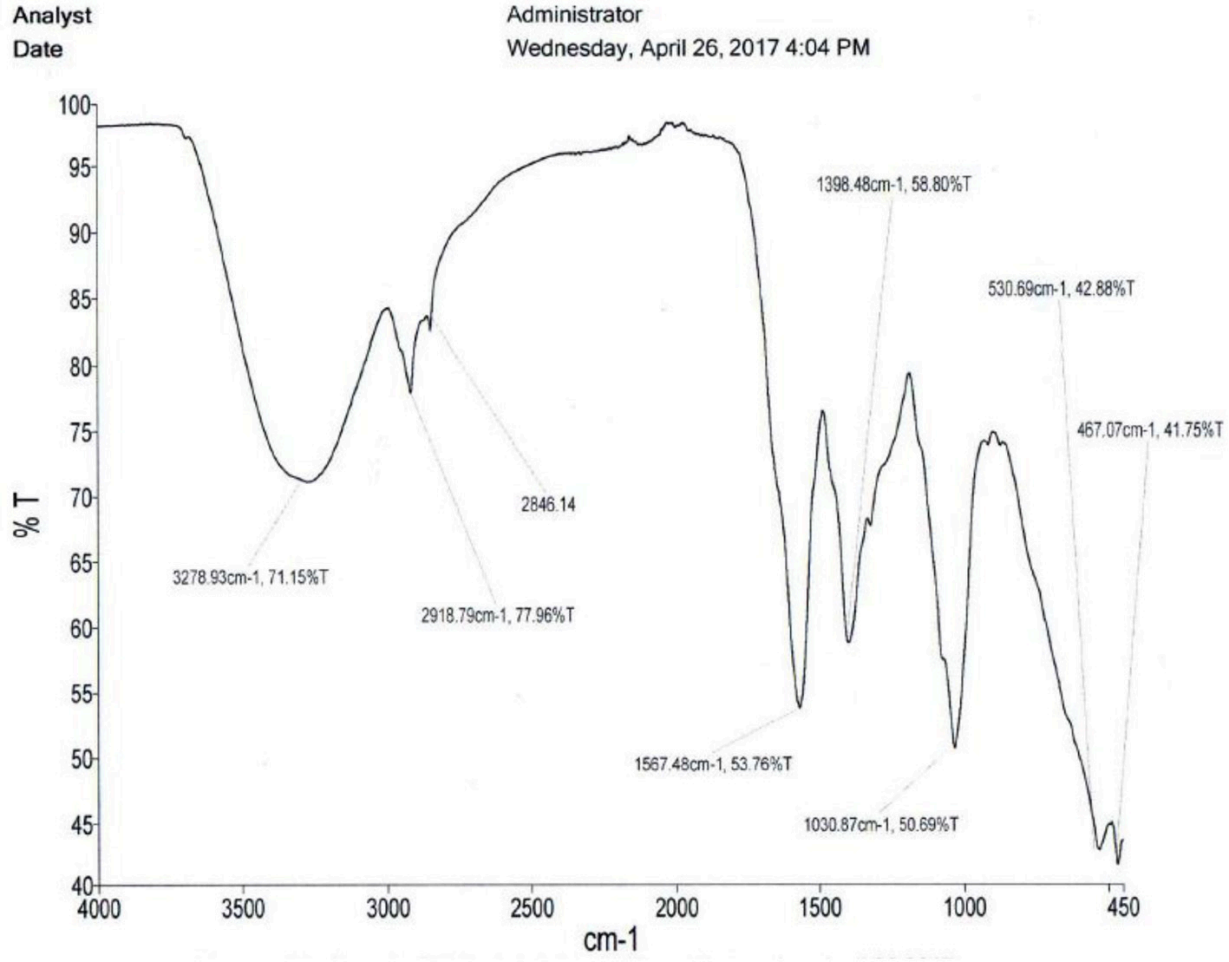
*Adenium multiflorum* extract spectrum.

**Figure 4:**
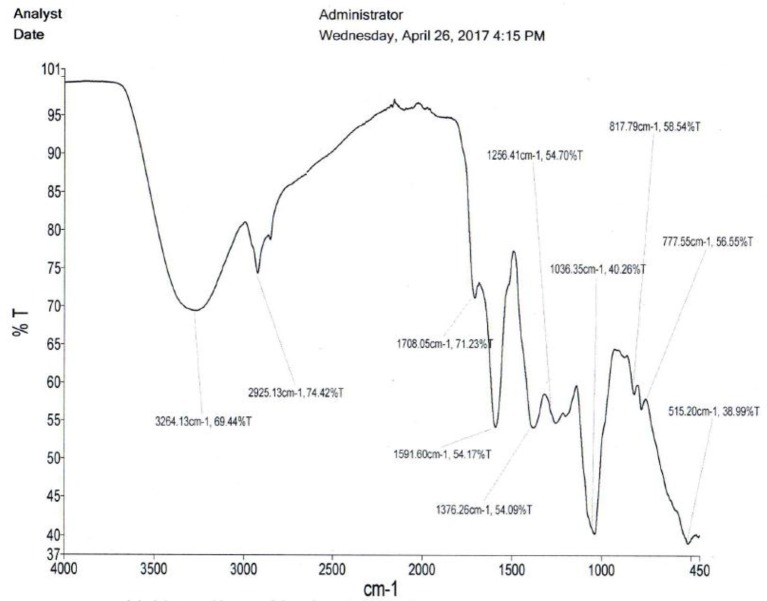
*Cissus quadrangularis* spectrum.

**Figure 5:**
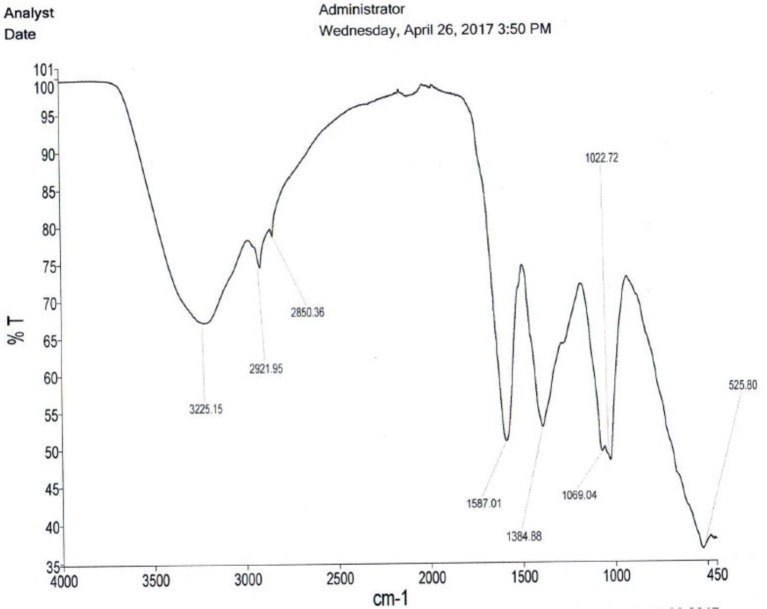
*Erythrina abyssinica* leaves spectrum.

Figures 3–5 are infrared spectra of the clean crude extracts of *A. multiflorum, C. quadrangularis* and *E. abyssinica* leaves done using PerkinElmer Spectrum Version 10.5.2. These are some of the unique ways of fingerprinting and identification of plants and/or their extracts for quality control and assurance as well as allowing duplication.

## Conclusion

Of all the plant extract ointments, *Cissus quadrangularis* ointment exhibited the best wound healing properties followed by *Adenium multiflorum* ointment. *Erythrina abyssinica* leaf and bark ointments exhibited wound healing properties similar to the negative control though the leaf ointment exhibited slightly better healing properties.

## Author contributions

All authors listed have made a substantial, direct and intellectual contribution to the work, and approved it for publication. AM conceptualized the ideas and planned the research. TM, SKh, AN, and GM supervised the work. SK, IM, and TM-T assisted with data collection and execution of research.

### Conflict of interest statement

The authors declare that the research was conducted in the absence of any commercial or financial relationships that could be construed as a potential conflict of interest.
